# Innovative use of the octopus stabilizer in the excision of a cardiac hydatid cyst

**DOI:** 10.1093/jscr/rjw019

**Published:** 2016-02-26

**Authors:** Mohammud Musleh, Nadia Abuhussein, Ghassan Musleh, Paul Waterworth

**Affiliations:** 1 School of Medicine, University of Liverpool, Liverpool, UK; 2 Peninsula Schools of Medicine and Dentistry, Plymouth University, Plymouth, UK; 3 Northwest Heart Centre, University Hospitals of South Manchester, Manchester, UK

## Abstract

Hydatid disease is caused through *Echinococcus granulosus* infection. Hydatid disease remains endemic in developing countries. The majority of cases involve the lungs or liver. We report the case of a patient diagnosed with concurrent mediastinal and cardiac cysts. In this patient, the Octopus IV cardiac stabilizer was used to rotate the heart after the excision of the mediastinal cyst, enabling the excision of a cyst adherent to left ventricle through a single median sternotomy incision. To date, there have been no reports of the application of the Octopus IV cardiac stabilizer in such a way.

## INTRODUCTION

Hydatid disease (cystic echinococcosis) is caused by infection with parasitic tapeworms (cestode), most commonly *Echinococcus granulosus* or *Echinococcus alveolaris*. It is endemic in developing regions such as Turkey, India and Africa [1]. Dogs and related species (such as foxes) are the definitive host. Tapeworm eggs are passed into dog faeces and ingested by intermediate hosts, including humans. Eggs may also spread to humans by physical contact [2].

Hydatid disease may present result in unilocular or multilocular cysts. These typically affect several organs, predominantly the liver and lungs. Cardiac hydatid cysts are present in fewer than 0.5% of cases [3], and mediastinal cysts are similarly rare [4]. Clinical features may be vague, and diagnosis depends on a high degree of clinical suspicion, particularly if the patient comes from an endemic area. Early management is essential due to important surrounding structures [4]. Surgical removal of the germinative membrane and pericyst through a suitable thoracic incision is required [5].

In this report, we present the case of a patient diagnosed with concurrent mediastinal and cardiac cysts. Treatment was surgical with a novel application of the Octopus IV cardiac stabilizer to excise both cysts using a single sternotomy incision.

## CASE REPORT

A 33-year-old man was referred to the thoracic surgery department complaining of breathing difficulties and a choking sensation when lying flat. He complained of no other symptoms. The patient provided a past history of a right thoracotomy for the excision of a hydatid cyst from his right lung. Physical examination was unremarkable.

**Figure 1:**
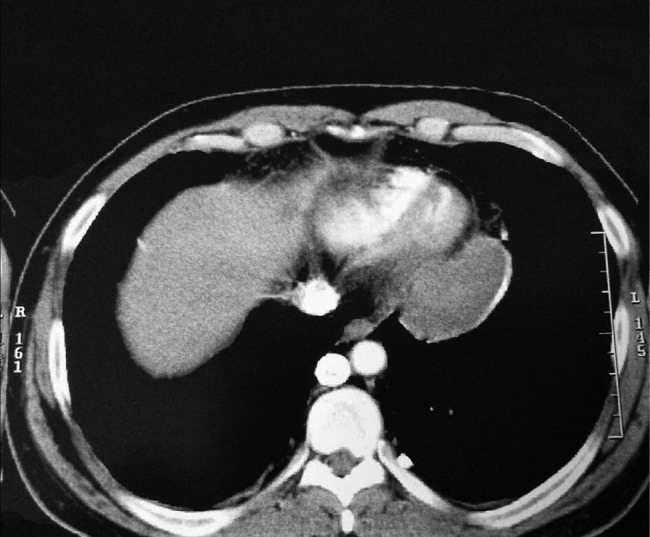
Chest CT with contrast showing mediastinal cysts.

Investigations initially showed a normal full blood count and a positive Casoni test. A chest CT scan with contrast subsequently revealed two multi-loculated mediastinal cysts. The first was located in the middle mediastinum lateral to the ascending aorta. The second was posterior to the heart attached to the posterolateral surface of the left ventricle (Fig. 1). Echocardiography demonstrated that the posterior cyst was adherent to the posterior surface of the left ventricle.

**Figure 2:**
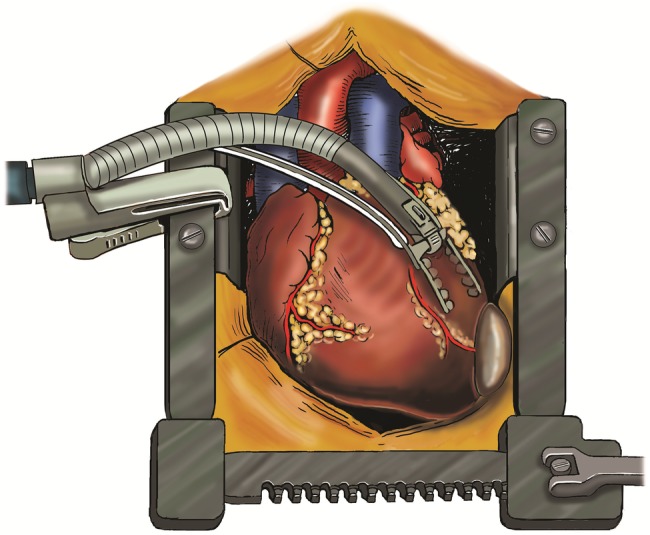
Illustration of the Octopus IV cardiac stabilizer positioning the heart, enabling the excision of the second hydatid cyst without an additional incision. The authors thank Helen Carruthers from the Department of Medical Illustrations at the University Hospital of South Manchester for the creation of this illustration.

**Figure 3:**
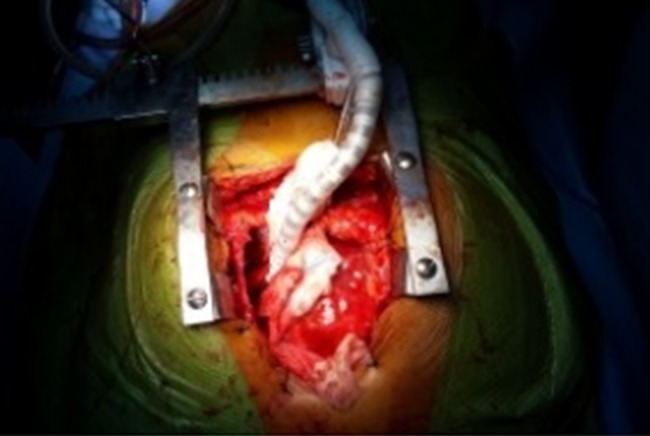
Intraoperative photograph of the Octopus IV cardiac stabilizer maintaining the position of the heart to expose the posterior hydatid cyst.

The cysts were approached through a median sternotomy incision. The pericardium was dissected free of the heart and major vessels. The cyst lateral to the aorta was excised first. Hypertonic (3.0%) saline was injected and the cyst fully excised. The anterior surface of the ectocyst was also excised. In order to expose the posterior cyst, the heart was lifted up, rotated to the right and maintained in this position by the Octopus IV cardiac stabilizer (Figs 2 and 3). The cyst was dissected free of the pericardium but remained adherent to the myocardium. The edges of the cyst were packed with small gauze soaked with hypertonic saline. Then, the cyst was injected with hypertonic saline. After 5 min, the cyst was opened and fully excised. The floor (posterior surface) of the cyst was widely excised leaving the residual cavity widely opened to the pericardial sac. Size 28F chest drains were inserted in the residual cavities, and the wound was closed in the routine way. The patient progressed well and was discharged home on the fourth postoperative day.

## DISCUSSION

The Octopus IV cardiac stabilizer (Medtronic, Watford, UK) consists of two suction footplates placed parallel to the target area. It utilizes this suction pressure to immobilize the target site. This low traumatic stabilization offers superior myocardial protection when compared with traditional methods of stabilization [6]. Waterworth *et al.* (2004) used the Octopus IV cardiac stabilizer to immobilize the right ventricular outflow tract to facilitate repair of a cardiac stab wound [7].

In this report, we describe the use of the Octopus IV cardiac stabilizer to safely lift the heart up to expose a hydatid cyst that was firmly adherent to the posterior surface of the heart and facilitate its excision. This enabled the excision of both the mediastinal and cardiac cyst without an additional incision.

## CONFLICT OF INTEREST STATEMENT

None declared.
